# The functions of the multi-tasking Pfh1^Pif1^ helicase

**DOI:** 10.1007/s00294-016-0675-2

**Published:** 2017-01-04

**Authors:** Nasim Sabouri

**Affiliations:** Department of Medical Biochemistry and Biophysics, Umeå University, 901 87 Umeå, Sweden

**Keywords:** Helicase, Pif1, Pfh1, *Schizosaccharomyces pombe*, G-quadruplex DNA, Telomere

## Abstract

Approximately, 1% of the genes in eukaryotic genomes encode for helicases, which make the number of helicases expressed in the cell considerably high. Helicases are motor proteins that participate in many central aspects of the nuclear and mitochondrial genomes, and based on their helicase motif conservation, they are divided into different helicase families. The Pif1 family of helicases is an evolutionarily conserved helicase family that is associated with familial breast cancer in humans. The *Schizosaccharomyces pombe* Pfh1 helicase belongs to the Pif1 helicase family and is a multi-tasking helicase that is important for replication fork progression through natural fork barriers, for G-quadruplex unwinding, and for Okazaki fragment maturation, and these activities are potentially shared by the human Pif1 helicase. This review discusses the known functions of the Pfh1 helicase, the study of which has led to a better understanding of nucleic acid metabolism in eukaryotes.

## Introduction

**Fig. 1:**
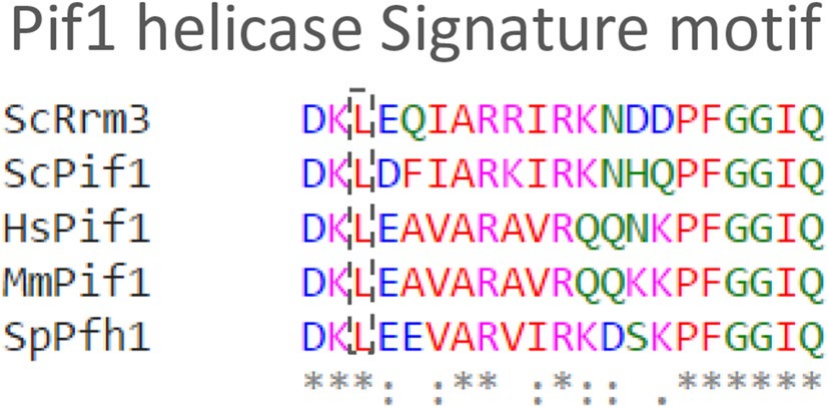
Alignment of the unique 21 amino acid Pif1 signature motif with sequences from *S. cerevisiae* Rrm3 (ScRrm3), *S. cerevisiae* Pif1 (ScPif1), *S. pombe* Pfh1 (SpPfh1), *Mus musculus* Pif1 (MmPif1), and *Homo sapiens* Pif1 (HsPif1). The alignment was performed in Clustal Omega (Sievers et al. 2011). The leucine variant detected in breast cancer families and the position of the corresponding amino acid is marked with a *dashed* *box*. *Marks positions with a conserved residue, “:” shows conservation between amino acid groups with similar properties, and “.” indicates conservation between amino acids that have low similarities. *Red* is used for hydrophobic residues A, V, F, P, M, I, L, and W; *blue* is used for acidic residues D and E; *magenta* is used for basic residues R and K; and *green* is used for the other residues S, T, Y, H, C, N, G, and Q

Helicases are molecular motor enzymes that play important roles in nucleic acid metabolism. They use the energy of ATP hydrolysis to separate double-stranded nucleic acid molecules or to remodel nucleic acid–protein complexes. Helicases are involved in various cellular functions, and many helicases are intimately associated with human diseases (Bochman 2014; Brosh 2013). Based on the sequence conservation of the helicase motifs, helicases are divided into six different superfamilies (Superfamilies 1–6) (Singleton et al. 2007). These families are further divided into two groups, A and B, based on the directionality of their translocation on nucleic acids. Pif1 helicases belong to the superfamily 1B helicases and translocate in the 5′–3′ direction on nucleic acids (Bochman et al. 2010). The Pif1 family of helicases is evolutionarily conserved from yeasts to humans (Bochman et al. 2010) and is also found in some prokaryotic genomes (Bochman et al. 2011; Liu et al. 2015; Zhou et al. 2016). They function both in nuclear and mitochondrial maintenance, and they are found in single or multiple copies in different organisms. For example, the *Saccharomyces cerevisiae* genome encodes for two Pif1 homologs, while the *Schizosaccharomyces pombe* and human genomes encode for only one. All Pif1 helicases have a highly conserved helicase domain, but their N- and C-terminal regions can differ significantly. In addition to the conserved helicase domains, Pif1 helicases also possess a 21 amino acid signature motif located between motifs II and III that is unique to the Pif1 family of helicases (Fig. 1) (Bochman et al. 2010). Human families with a predisposition for breast cancer carry a mutant gene encoding an L319P variant at a highly conserved location in the 21 amino acid signature motif of Pif1 (Fig. 1) (Chisholm et al. 2012). However, how this motif is responsible for disease is largely unknown.

The *S. pombe* Pif1 homologue, Pfh1, shares 36% sequence identity with the conserved motifs in the helicase domain of human PIF1 (hPIF1) (Zhou et al. 2000) and is essential for maintaining the nuclear and mitochondrial genomes (Pinter et al. 2008). *S. pombe* cells carrying the corresponding breast cancer mutation, *pfh1*-*L430P*, are inviable (Chisholm et al. 2012). In addition, the Pfh1-L430P variant cannot perform the essential functions of wild-type Pfh1 in the mitochondria or the nucleus, suggesting that this motif or this specific position performs a critical function in Pif1 helicases (Chisholm et al. 2012).

*S. pombe* is a genetically tractable model organism, and has a similar genomic organization as human cells (Hoffman et al. 2015). This review highlights what is known about the functions of Pfh1—the *S. pombe* Pif1-family helicase—and compares this to what is known about other Pif1 helicases such as the poorly studied human Pif1 helicase (hPif1) and the well-studied *S. cerevisiae* Pif1 helicases (ScPif1 and ScRrm3).

## Pfh1 interacts with the replisome and plays a role in Okazaki fragment maturation

Replication of the nuclear double-stranded DNA is semi-conservative and occurs continuously on the leading strand and discontinuously on the lagging strand. The replisome consists of many different proteins, and some are needed on both strands while others are more strand-specific. Pfh1 translocates in the 5′–3′ direction on DNA (Tanaka et al. 2002; Zhou et al. 2002), but it is still not clear whether Pfh1 functions on both strands or if it is a strand-specific helicase.

Pfh1 interacts with many of the core proteins of the replisome, including the catalytic subunit of the leading-strand polymerase DNA polymerase ε, Pol2, the processivity clamp PCNA, the replicative helicase MCM complex, the single-stranded DNA-binding protein RPA, and the nuclease Dna2 (McDonald et al. 2016). Pfh1 and Pol2 are both enriched in the same regions during DNA synthesis, suggesting that they are in close proximity to each other during DNA replication (McDonald et al. 2016).

The discontinuous Okazaki fragments on the lagging strand must be ligated together to create a continuous DNA strand. The first step in the process is the removal of the RNA primer that is needed for the initiation of each fragment, and this is followed by subsequent ligation of the Okazaki fragments. This process requires DNA polymerase δ, the Dna2 and Fen1 nucleases, and DNA ligase I. A genetic study suggests that Pfh1 also plays a role in Okazaki fragment maturation on the lagging strand because a loss-of-function *pfh1*-*R20* mutant can rescue the cell growth of the heat-sensitive *dna2*-*C2* mutant at 37 °C (Ryu et al. 2004). The Dna2 nuclease is encoded by an essential gene, and this nuclease degrades long flaps that have eluded Fen1 cleavage during Okazaki fragment maturation. It is proposed that these long flaps are made by DNA polymerase δ and Pfh1 during excessive strand displacement and that Pfh1 is needed at these flaps to perhaps resolve DNA secondary structures that would otherwise inhibit the nuclease activity of Dna2 (Ryu et al. 2004). A similar function in Okazaki fragment maturation is suggested for the ScPif1 helicase of *S. cerevisiae* (Budd et al. 2006; Pike et al. 2009; Rossi et al. 2008).

## Pfh1 unwinds G-quadruplex DNA structures

G-quadruplex (G4) DNA is a four-stranded structure formed by stacked G-tetrads. G4 structures are stable and form in certain G-rich sequences, and if these remain unresolved in the genome they can act as obstacles to DNA replication (Mendoza et al. 2016). However, G4 structures have also been implicated in important biological functions such as transcription regulation, origin firing, and telomere maintenance (Rhodes and Lipps 2015). To reveal their specific biological functions, it is necessary to predict and map the occurrence of G4 structures in vivo. When searching for the sequence motif (G_3_N_1–25_)_3_G_3_, about 450 sequences are predicted to form G4 structures (such sequences are hereafter called G4 motifs) in the *S. pombe* genome (Sabouri et al. 2014). These G4 motifs are not randomly placed in the genome, but similar to humans they are enriched at telomeres, ribosomal DNA (rDNA), nucleosome-depleted regions, and promoters (Chambers et al. 2015; Hanakahi et al. 1999; Hansel-Hertsch et al. 2016; Huppert and Balasubramanian 2007; Parkinson et al. 2002; Sabouri et al. 2014) and have been implicated in origin firing (Besnard et al. 2012; Kanoh et al. 2015). Two of the predicted G4 structures in the *S. pombe* genome, one within an rDNA sequence and one within a telomeric sequence, have been well studied in vitro (Wallgren et al. 2016). A Taq DNA polymerase stop assay showed that the G4 motif from *S. pombe* rDNA arrests DNA synthesis by the Taq polymerase two nucleotides prior to the G4 motif (Jamroskovic et al. 2016), suggesting that formation of the rDNA G4 structure in the genome results in an obstacle to DNA synthesis.

In all organisms examined to date, Pif1 helicases act as potent G4 structure unwinders (Duan et al. 2015; Liu et al. 2015; Mendoza et al. 2015; Paeschke et al. 2013; Sanders 2010; Wallgren et al. 2016; Zhou et al. 2016) and as suppressors of the genomic instability that is observed in and around G4 motifs (Lopes et al. 2011; Paeschke et al. 2011; Ribeyre et al. 2009; Sabouri et al. 2014). In *S. pombe*, it was demonstrated by chromatin immunoprecipitation combined with sequencing (ChIP-seq) that Pfh1 binds to 20% of all G4 motifs (Sabouri et al. 2014). In cells depleted of Pfh1, fork pausing (measured as Pol2 occupancy) and DNA damage (measured as γ-H2A occupancy) is increased at G4 motifs, suggesting that Pfh1 is needed at these sites to facilitate DNA replication (Sabouri et al. 2014). In vitro, nuclear Pfh1 binds to and unwinds both intermolecular and intramolecular G4 structures (Wallgren et al. 2016). Together, these data suggest that unresolved G4 structures cause replication fork pausing in *S. pombe* cells and that one of Pfh1’s roles is to unwind G4 structures ahead of the replication fork.

## Pfh1 promotes replication at hard-to-replicate sites

Similar to hPif1 (Zhang et al. 2006), ScPif1, and ScRrm3 (Ivessa et al. 2002; Zhou et al. 2000), Pfh1 is enriched at telomeres in vivo (McDonald et al. 2014), and thus it is likely that it plays a direct role in the function of telomeres. In vitro, nuclear Pfh1 binds to a telomeric DNA substrate consisting of GGGTTACA telomeric repeats (Wallgren et al. 2016). Because *pfh1* ^+^ is an essential gene (Tanaka et al. 2002; Zhou et al. 2002), spore clones from *pfh1Δ* strains divide only 1–3 times, and these strains show stable but shorter telomeres than wild-type cells (Zhou et al. 2002). Also, overexpression of Pfh1 shows telomere lengthening (McDonald et al. 2014), suggesting that Pfh1 is a positive regulator of telomere length. In contrast to Pfh1’s role as a positive regulator of telomere length, hPif1, ScPif1, and ScRrm3 have been shown to be negative regulators of telomere length and to regulate telomerase activity because ScPif1 mutant cells and ScRrm3-deleted cells exhibit increased telomere length (Ivessa et al. 2002; Phillips et al. 2015; Schulz and Zakian 1994; Zhou et al. 2000) and overexpression of ScPif1 and hPif1 cause telomere shortening (Zhang et al. 2006; Zhou et al. 2000). This suggests that *S. pombe* cells use another helicase or another mechanism to regulate telomerase at telomeres and at sites of DNA breaks. The positive regulation of telomere length by Pfh1 might be due to Pfh1’s role in facilitating DNA replication at telomeres. In support of this, two-dimensional (2D) gel analysis of replication intermediates has shown that cells depleted of Pfh1 exhibit increased pausing at telomeres (McDonald et al. 2014), suggesting that Pfh1 facilitates fork progression at telomeres.

How Pfh1 facilitates replication at telomeres is still unknown. However, because a telomeric DNA sequence from *S. pombe* forms an intramolecular hybrid G4 structure in vitro and Pfh1 can unwind this structure (Wallgren et al. 2016), the requirement for Pfh1 at telomeres during replication fork progression might be due to Pfh1’s ability to unwind G4 structures. Pfh1 might also facilitate fork progression at telomeres by removing non-nucleosomal proteins from the telomeres; however, it is not known if these proteins block replication fork progression at telomeres.

DNA damage hotspots are found at RNA polymerase III transcribed genes in temperature-sensitive *pfh1* mutant strains of *S. pombe*, suggesting that Pfh1 is needed at these sites to suppress DNA damage (Zhou et al. 2013). Also, 2D gel analysis has shown that fork progression in *S. pombe* is dependent on Pfh1 at several highly transcribed RNA polymerase III genes, the tRNA and 5S rRNA genes, and the highly transcribed RNA polymerase II genes *act1* ^+^, *hta1* ^+^, and *htb1* ^+^ (Sabouri et al. 2012). In addition, ChIP-seq experiments have shown that Pfh1 is enriched at approximately 50% of all tRNA and 5S rRNA genes and at 60% of the top 500 highly transcribed RNA polymerase II genes (McDonald et al. 2016). These results have been confirmed by the observation that Pfh1-depleted cells exhibit increased fork pausing and DNA damage at these sites, suggesting that Pfh1 is needed at these sites to facilitate fork progression (McDonald et al. 2016). ScRrm3 is also needed at highly transcribed RNA polymerase III genes to promote replication (Ivessa et al. 2003), but to date Pfh1 is the sole Pif1-family helicase that has been shown to be necessary at highly transcribed RNA polymerase II genes. Similar to ScRrm3, Pfh1 is needed to promote the merging of converging forks (Ivessa et al. 2000; Sabouri et al. 2012; Steinacher et al. 2012).

To allow for high rates of transcription of rDNA and for efficient mating type switching at mating type loci, replication of these regions is unidirectional, and this unidirectionality is caused by the binding of the non-histone proteins, such as Swi1, Swi3, and Sap1 that act as replication fork barriers at specific sites within these regions (Arcangioli et al. 1994; Dalgaard and Klar 2000, 2001; Krings and Bastia 2004, 2005; Mejia-Ramirez et al. 2005). By 2D gel analysis, it has been shown that Pfh1 is needed to facilitate replication through these barriers at both rDNA and mating type loci (Sabouri et al. 2012; Steinacher et al. 2012). In the absence of the Timeless homolog Swi1 that tightly binds these sites, replication at rDNA and mating type loci is no longer dependent on Pfh1, suggesting that Pfh1 removes Swi1 from the barriers (Sabouri et al. 2012).

All of the above-mentioned regions, including telomeres, highly transcribed genes, and replication fork barriers, are stably bound by protein complexes and thus are hard-to-replicate sites. Therefore, one possible function for Pfh1 at these sites might be to remove these proteins from DNA so that these tightly bound proteins do not block DNA replication. Another function for Pfh1 might be to resolve R-loops within the highly transcribed genes that would otherwise hinder replication. However, these activities for Pfh1 have not been examined in vitro.

## Pfh1 localizes to mitochondria and interacts with mitochondrial proteins

Immunofluorescence microscopy has shown that Pfh1 localizes to the mitochondria (Pinter et al. 2008). Moreover, depletion of Pfh1 causes loss of mitochondrial DNA number, and *S. pombe* cells with the *pfh1*-*m1* allele (coding for an isoform that localizes only to the nucleus) are inviable, showing that Pfh1 is essential for mitochondrial maintenance (Pinter et al. 2008). In addition, affinity purification of Pfh1 combined with mass spectrometry has shown that Pfh1 interacts with several known mitochondria-localizing proteins, such as the mitochondrial single-stranded binding protein Rim1, the mitochondrial repair protein Mgm101, and the mitochondrial RNA polymerase Rpo41 (McDonald et al. 2016). Together, these data support the notion that Pfh1 functions in the mitochondrial DNA. However, what these functions of Pfh1 are in the mitochondria is still not known.

Pif1 helicases from other organisms also play a role in mitochondria. For example, ScPif1 was actually first discovered as a mitochondrial protein that affected mitochondrial recombination frequency (Foury and Kolodynski 1983), and mitochondrial myopathy appears in *pif1* knockout mice (Bannwarth et al. 2016).

As previously mentioned, G4 DNA structures are built of stacked G-tetrads, and nucleic acids with two or more stacks of G-tetrads can form a G4 structure in vitro. The most common G4 structures studied in the nuclear genome are G4 structures with stacks of three G-tetrads because these are more stable than G4 structures with stacks of two G-tetrads. The *S. pombe* mitochondrial genome does not contain any G4 motifs with three G-tetrad stacks. However, when scanning the mitochondrial genome for G4 motifs with two G-tetrads stacks, 6 and 45 G4 motifs were found when using (G_2_N_1–7_)_3_G_2_ and (G_2_N_1–20_)_3_G_2_, respectively, in the search algorithms (Table 1). It is not known, however, if these G4 motifs form G4 structures, and the association of Pfh1 with these sites has not been examined. However, it is tempting to speculate that one of the functions of Pfh1 in the mitochondrial DNA is to resolve G4 structures just as it does in the nuclear DNA.

**Table 1:** G4 motifs identified in the mitochondrial genome using the (G_2_N_1–7_)_3_G_2_ algorithm

Start coordinate	End coordinate	Forward/reverse strand	Sequence
4967	4989	F	GGATTGGTATCTGGGATAATTGG
5267	5288	F	GGACCTGGTGGTGGTTGGACGG
17851	17879	F	GGTGTTAGTGGTGCTGGTGTTGGTATTGG
4652	4676	R	CCCTTTTACCAACTTTTCCTTAACC
11049	11074	R	CCCGAATTCCAATTCCCATCTCACCC
18118	18141	R	CCAGAATCTCCATTTTCCCCCTCC

The sequence of the forward strand is shown

## Pfh1 in DNA repair

In addition to the nuclear and mitochondrial isoforms of Pfh1, a third isoform of Pfh1 can be detected when *S. pombe* cells are exposed to the DNA damaging agent camptothecin (Pinter et al. 2008). In addition, Pfh1 co-localizes with DNA damage foci (Pinter et al. 2008), and *S. pombe* strains with temperature-sensitive mutations in *pfh1* are sensitive to the DNA damaging agents hydroxyurea and methyl methane sulfonate (Tanaka et al. 2002). Together, these data suggest that nuclear Pfh1 is needed during DNA damage repair. This conclusion is strengthened by affinity purification experiments combined with mass spectrometry that demonstrate that Pfh1 interacts with several proteins involved in DNA repair, such as the mismatch repair proteins, the ATR checkpoint kinase Rad3, and the DNA recombination protein Rad52 (McDonald et al. 2016).

## Conclusions

*S. pombe* is a great model organism when studying chromosome biology, as its chromosomal organization resembles the one in human cells (Olsson and Bjerling 2011). Pfh1 from *S. pombe* exhibits the combined functions of the two most studied Pif1 helicases, ScPif1 and ScRrm3, but it also has some functions that have not been shown for other Pif1-family helicases. For example, it plays a role as a positive regulator of telomere length, and it promotes replication of highly transcribed RNA polymerase II genes. Because *S. pombe* and humans only encode one Pif1 helicase, it is very likely that hPif1 has many similar functions in the cell as Pfh1. These functions and perhaps some yet undiscovered functions of Pif1 helicases will be important to study in the future to understand the role of hPif1 in breast cancer and potentially other diseases.
